# Selective RET inhibitors shift the treatment pattern of *RET* fusion-positive NSCLC and improve survival outcomes

**DOI:** 10.1007/s00432-022-04188-7

**Published:** 2022-07-15

**Authors:** Chang Lu, Xue-Wu Wei, Yi-Chen Zhang, Zhi-Hong Chen, Chong-Rui Xu, Ming-Ying Zheng, Jin-Ji Yang, Xu-Chao Zhang, Qing Zhou

**Affiliations:** 1 The Second School of Clinical Medicine, Southern Medical University, Guangzhou, 510515 China; 2 Guangdong Lung Cancer Institute, Guangdong Provincial People’s Hospital, Guangdong Academy of Medical Sciences, Guangzhou, 510080 China

**Keywords:** Non-small cell lung cancer, *RET* fusion, Real-world data, Tyrosine kinase inhibitor, Adverse event

## Abstract

**Purpose:**

Rearranged during transfection (RET) fusions are important genetic drivers in non-small cell lung cancer (NSCLC). Selective RET inhibitors are setting a new paradigm in *RET*-driven NSCLC. However, the real-world treatment patterns, outcomes and toxicity remain largely unknown.

**Methods:**

Data from *RET* fusion-positive NSCLC patients treated in our centre were retrospectively analysed. Of them, patients diagnosed before and after August 2018 were included in analysis of treatment patterns; and patients received selective RET inhibitors were eligible for analysis of adverse events (AEs).

**Results:**

Patients diagnosed before August 2018 (*n* = 30) predominantly received chemotherapy and immunotherapy (83%) as initial therapy, while patients diagnosed after August 2018 (*n* = 39) mainly received selective RET inhibitors (38.5% at first-line; 50.0% at second-line). In the total 69 patients, overall survival (OS) was prolonged in patients treated with selective RET inhibitors versus untreated patients (median 34.3 versus 17.5 months; *p* = 0.002) during a median follow-up of 28.7 months. But there was no difference between patients treated with immunotherapy versus untreated patients. In the 38 patients received selective RET inhibition, median progression-free survival (PFS) was 11.9 months. AEs ≥ grade 3 occurred in 42.1% patients and were not associated with PFS (p = 0.63) or OS (*p* = 0.60). Haematological toxicity ≥ grade 3 occurred in 31.6% patients and was the leading cause of drug discontinuation.

**Conclusion:**

Selective RET inhibitors are increasingly being adopted into clinical practice and are associated with improved OS. However, treatment-related ≥ grade 3 AEs, especially haematologic AEs, occur frequently in real-world setting.

## Introduction

Rearranged during transfection (RET) fusion are found in 1–2% of non-small cell lung cancers (NSCLCs) (Wang et al. 2012). It is recommended that the presence of *RET* fusion be determined routinely at diagnosis (Belli et al. 2021). Traditional multitarget kinase inhibitors (MKIs) (Gautschi et al. 2017; Drilon et al. 2016, 2019; Yoh et al. 2021; Lee et al. 2017; Takeuchi et al. 2020) have been used for the treatment RET fusion positive lung cancers, but they are associated with limited efficacy. In the past, the treatment algorithms of advanced *RET* fusion positive lung cancers have been similar to the treatment algorithms of oncogene-negative NSCLC (chemotherapy or immune checkpoint inhibitors, as monotherapies or in combination). This has recently begun to change with the development of selective RET tyrosine kinase inhibitors (TKIs), which are setting a new paradigm for personalized treatment. There are two selective RET inhibitors currently being approved by FDA: pralsetinib and selperctatinib. The findings from the Phase I/II, global, multicenter, registrational ARROW trial (ClinicalTrials.gov Identifier: NCT03037385) revealed a high objective response rate (ORR) and a sustained median progression-free survival (mPFS) of pralsetinib in both previously treated (61%; 17.1 months) and untreated patients (70%; 9.1 months) (Gainor et al. 2021; Ali et al. 2022). Similarly, according to the registrational dataset analysis of a phase I/II LIBRETTO-001 trial (ClincalTrials.gov identifier: NCT03157128), selpercatinib showed an ORR of 64% in pretreated RET fusion-positive NSCLC patients, and 85% in treatment-naïve patients (Drilon et al. 2020). However, as the treatment landscape evolves, there is limited direct evidence regarding the treatment patterns and outcomes of *RET* fusion-positive NSCLC patients.

Besides efficacy, it is imperative for physicians to be aware of the toxicity associated with therapies. Previous evidence has consistently shown that although both FDA Approved selective RET inhibitors are well tolerated, adverse events (AEs) are common in clinical trials. For pralsetinib, the most common any grade treatment-related AEs included elevated aspartate aminotransferase (AST) level (34%), anaemia (24%), elevated alanine aminotransferase (ALT) level (23%), and hypertension (22%); while for selpercatinib, the most observed the majority of treatment-related toxicities included diarrhoea (25%), increased AST (22%), increased ALT (20%), and hypertension (17%) (Drilon et al. 2020; Gainor et al. 2021; Ali et al. 2022). These AEs can result in dose reduction, treatment discontinuation and, in some cases, these reactions can be life-threatening. However, real-world evidence of outcomes of grade 3 or worse AEs occurring during treatment and their impact on overall survival remains unknown.

This study aimed to report real-world treatment patterns, survival outcomes and implications of toxicities of selective RET TKI in advanced *RET*-driven NSCLC patients.

## Materials and methods

### Patients and study selection

**Fig. 1:**
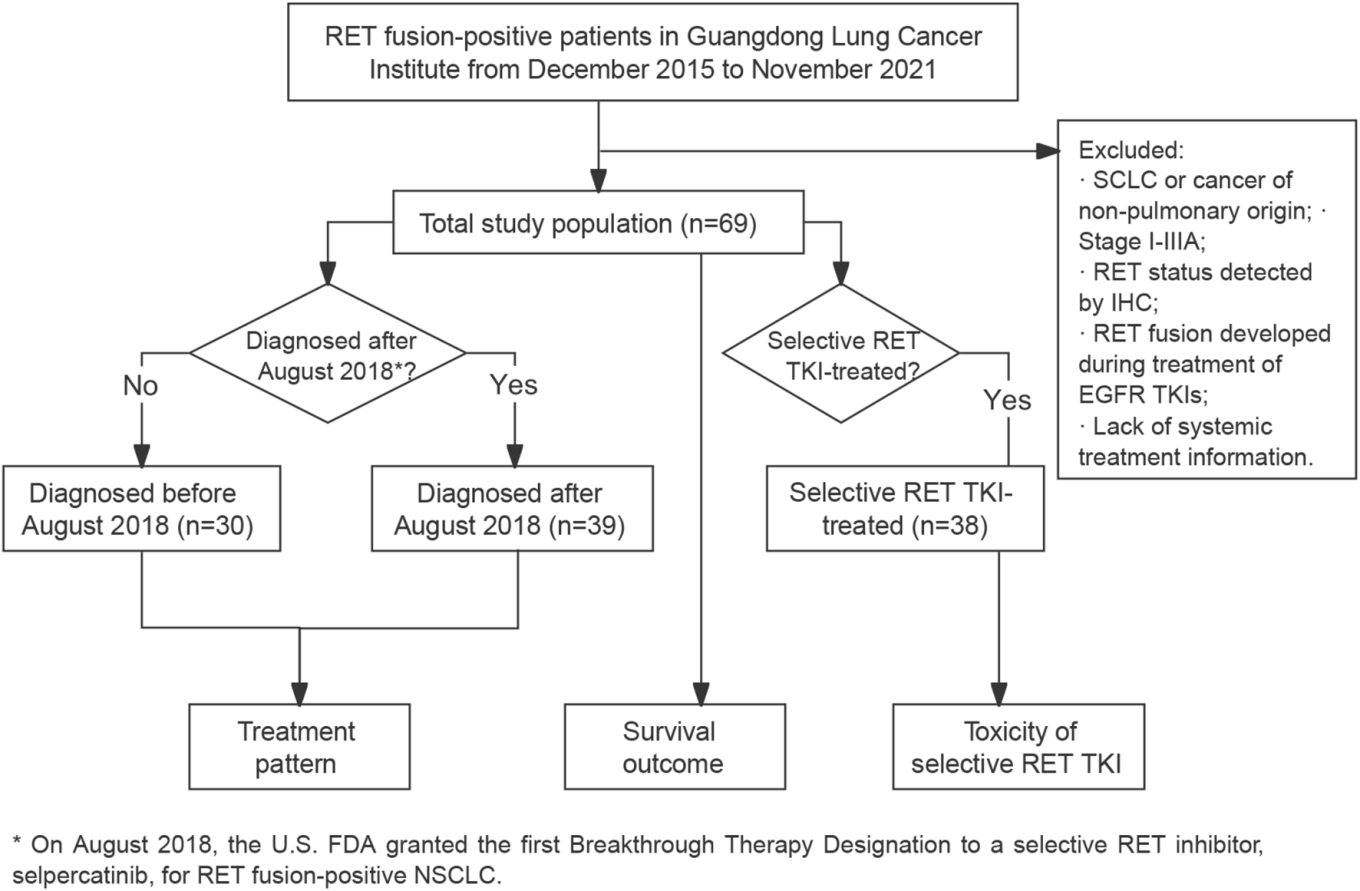
Study design and flowchart. Abbreviations: SCLC, small cell lung cancer; IHC, immunochemistry; TKI, tyrosine kinase inhibitor

Patients with *RET* fusion-positive solid tumours who had been treated at the Guangdong Lung Cancer Institute from December 2015 to November 2021 were screened for eligibility into the study. We enrolled patients with a pathologic diagnosis of locally advanced or metastatic NSCLC with a *RET* fusion diagnosed using either fluorescence in situ hybridization, reverse transcriptase polymerase chain reaction, or next-generation sequencing. Patients who developed *RET* fusion after exposure to EGFR TKIs were excluded. On August 2018, the U.S. FDA granted the first Breakthrough Therapy Designation to a selective RET inhibitor, selpercatinib, for *RET* fusion-positive NSCLC (Markham 2020). Therefore, we took August 1, 2018 as the date to compare real-world treatment patterns before and thereafter. The study was approved by the Research Ethics Committee of Guangdong Provincial People’s Hospital and all included patients provided written informed consent (the study flowchart is presented in Fig. 1).

### Data collection and outcome measures

Treatment and management of patients in our centre was in compliance with Good Clinical Practice. Clinical data including age, gender, *RET* upstream fusion partner, tumour stage, date of diagnosis, treatment history and death were recorded. Tumour response and progression was assessed using the Response Evaluation Criteria in Solid Tumours v1.1. Data collection cut-off was on January 18, 2022. Patients with information on systemic treatments were included in analysis of real-world treatment patterns; and patients who were treated with any selective RET inhibitor were eligible for analysis of AEs and clinical outcomes of selective RET inhibition.

Data on AEs and drug interruption, discontinuation and dosage reduction were also collected. AEs that occurred after the initiation of any selective RET inhibitor and prior to subsequent systemic anticancer treatment were graded per the National Cancer Institute Common Terminology Criteria for Adverse Events. Grade 3 or worse AEs were included for analysis.

Time to treatment failure (TTF) was measured as the time from the initiation of treatment with selective RET inhibitor to discontinuation, regardless of whether discontinuation was due to disease progression, treatment toxicity, or death. PFS was measured as the time from the initiation of treatment with selective RET inhibitor to disease progression or death, whichever occurred first. Overall survival was measured as the time from the initiation of first-line treatment to death from any cause.

### Statistical analysis

Data were summarized according to frequency and percentage for categorical variables as well as by medians and ranges for continuous variables. Survival rates were summarized and analysed using the Kaplan–Meier method with Log-rank test. Patients who had not experienced the required events at the time of data cut-off were defined as censored at their last follow-up. Reverse K-M method was used to calculate median follow-up time. The impact of treatment and metastatic sites on survival was evaluated using hazard ratios (HRs) and 95% CIs. Statistical analyses were carried out by using SPSS software (version 23.0, IBM SPSS Statistics).

## Results

### Treatment patterns of *RET* fusion-positive NSCLC patients

**Fig. 2:**
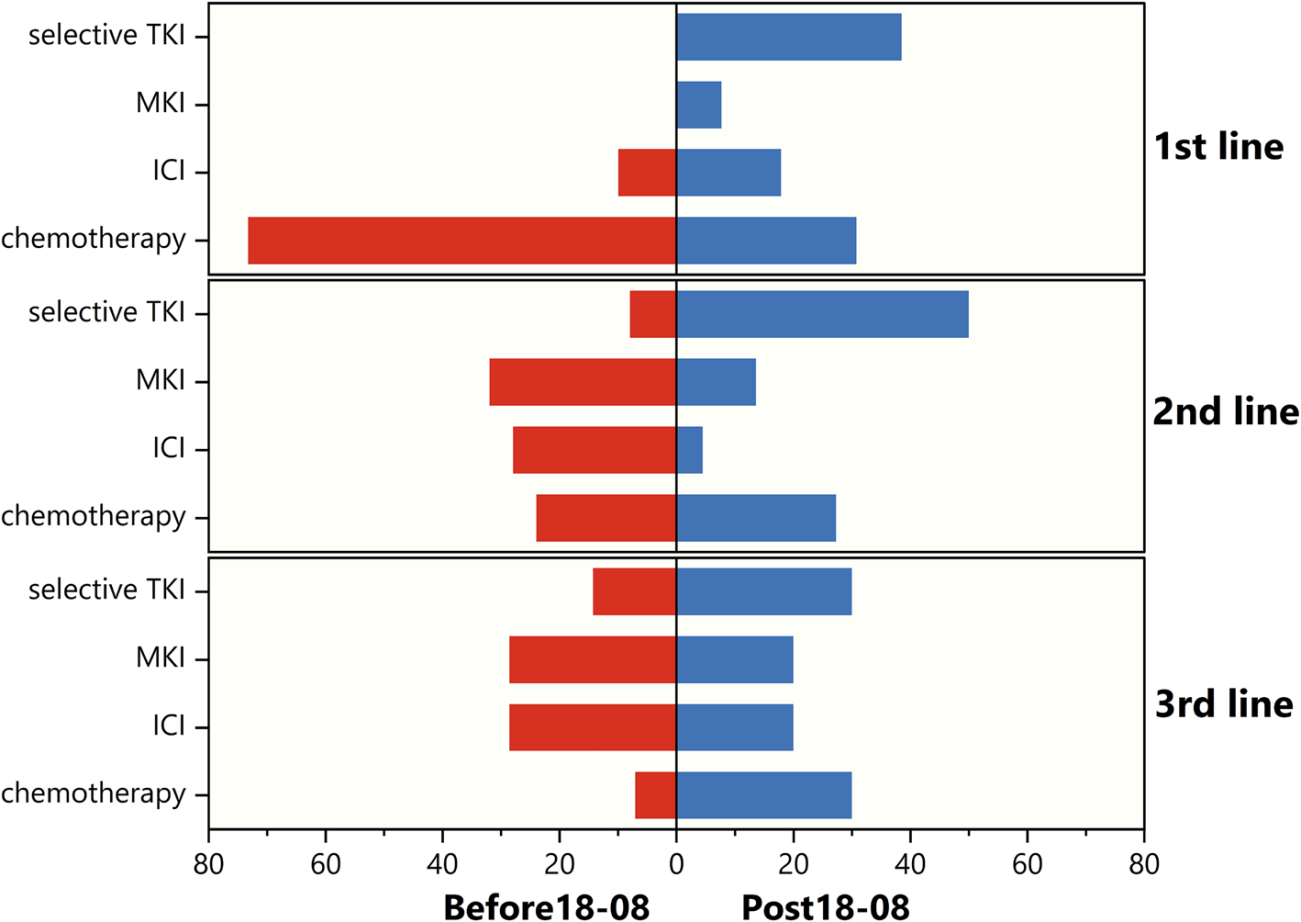
A paradigm shift in real-world treatment patterns of *RET*-driven NSCLC before and post August 2018 (the first FDA Breakthrough Therapy Designation to selpercatinib, a selective RET inhibitor). Treatment distributions in the context of different line settings. Abbreviations: MKI, multikinase inhibitor with activity against RET; ICI, immune checkpoint inhibitor; selective TKI, selective RET tyrosine kinase inhibitor

As mentioned in Methods, we compared real-world treatment patterns before and after August 1, 2018. Of the 69 patients included in the treatment pattern analysis, 30 (43.5%) and 39 (56.5%) were diagnosed before and after August 1, 2018, respectively. Most of the patients diagnosed before August 1, 2018 received chemotherapy (73.3%) as first-line (1L) treatment, and MKI (32.0% at 2L; 28.6% at 3L) or immunotherapy (28.0% at 2L; 28.6% at 3L) thereafter. Patients diagnosed after August 1, 2018 mainly received selective RET inhibitor (38.5% at 1L; 50.0% at 2L; 30.0% at 3L) and chemotherapy (30.8% at 1L; 27.3% at 2L; 30.0% at 3L). Analyses of 1/2/3L treatment distributions in the overall population is presented in Fig. 2. Patients diagnosed after August 1, 2018 received MKI treatment less frequently than patients diagnosed earlier (20.5% [8/39] vs. 40.0% [12/30]), and mostly received selective RET inhibitors (76.9% [30/39] vs. 26.7% [8/30]), with an enrichment in first- and second-line treatments. Interestingly, immunotherapy was also a therapeutic option in *RET*-driven NSCLC patients at any line of treatment, with immune checkpoint inhibitor (ICI) combinations being more common in 1L and ICI monotherapy more common in 2/3L.

### Treatment outcomes of different therapeutical regimens in *RET*-driven NSCLC patients

**Fig. 3:**
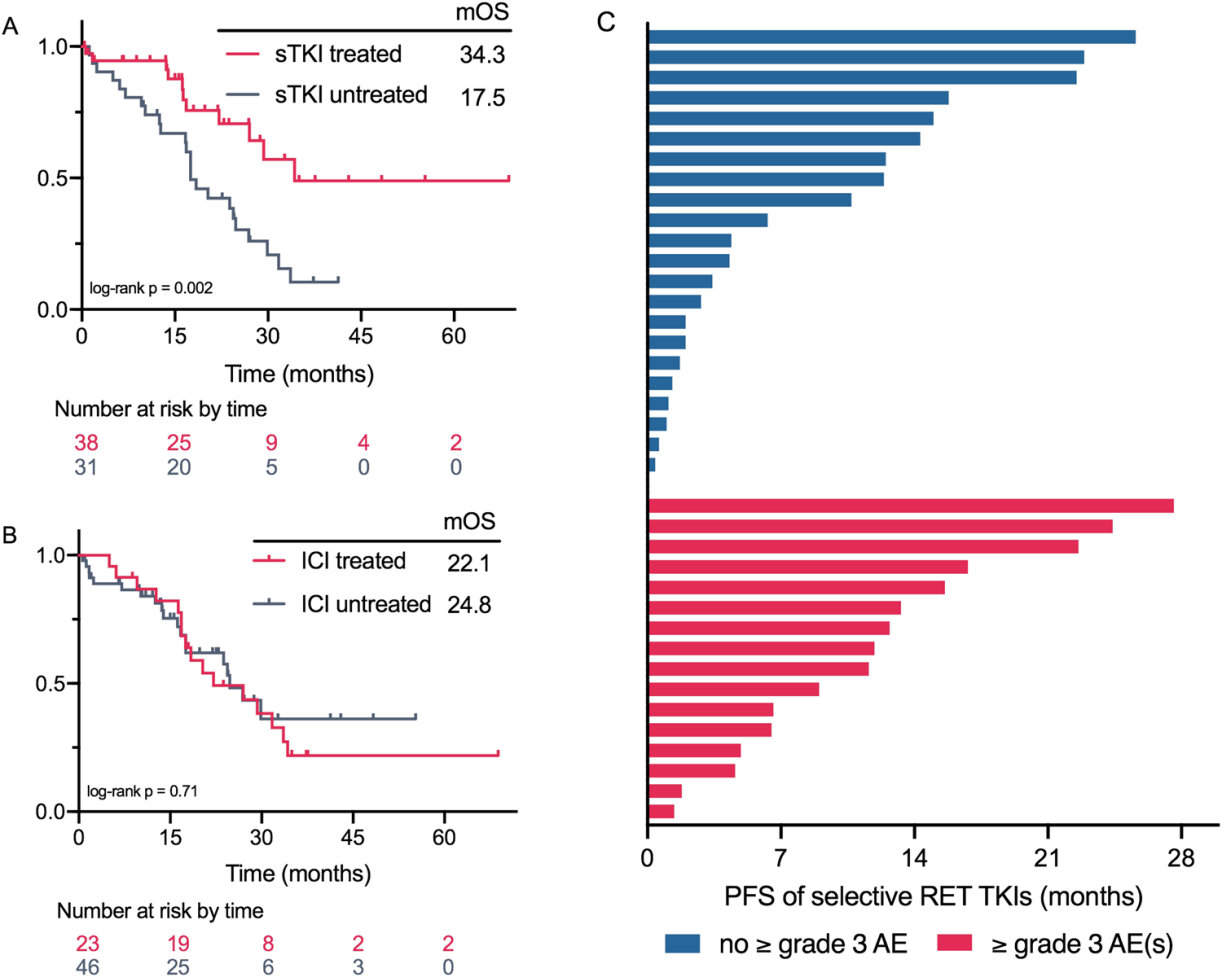
Survival outcomes of different therapeutical regimens in *RET*-driven NSCLC patients. Kaplan–Meier estimates of OS of patients receiving selective RET inhibitors (**A**) or immunotherapy (**B**) throughout the course of disease. **C** Swimmer plot of PFS of selective RET inhibitors in patients with and without ≥ grade 3 AEs. Abbreviations: OS, overall survival; sTKI, selective RET tyrosine kinase inhibitor; ICI, immune checkpoint inhibitor; PFS, progression-free survival; AE, adverse event

We included 69 patients from our institute in the analysis of clinical outcomes. Table 1 summarizes their clinicopathological characteristics. Of all patients, 46.4% were female, 56.5% were non-smoker and 69.6% harboured *KIF5B-RET* fusion. The median duration of follow-up was 28.7 months. Overall survival was prolonged in patients treated with selective RET TKIs when compared with untreated patients (median 34.3 versus 17.5 months; HR: 0.34, 95% CI: 0.18–0.68, *p* = 0.002; Fig. 3A), but there was no difference in those treated with MKIs throughout the course of disease (HR: 1.20, 95% CI 0.58–2.47, *p* = 0.63). Notably, there was no difference in OS between patients treated with ICIs and patients not treated with ICIs throughout the course of disease (median 22.1 months for ICI-treated, versus 24.8 months for ICI-naive; HR: 1.14, 95% CI 0.58–2.24, *p* = 0.71; Fig. 3B).

**Table 1:** Clinicopathological characteristics of *RET* fusion-positive patients

	Selective TKI naïve	Selective TKI treated	Total
	(*N* = 31)	(*N* = 38)	(*N* = 69)
*Follow-up time (months)*			
Median [95% CI]	28.7 [22.9, 41.3]		
*Sex*			
Female	12 (38.7%)	20 (52.6%)	32 (46.4%)
Male	19 (61.3%)	18 (47.4%)	37 (53.6%)
*Smoking history*			
Former	9 (29.0%)	8 (21.1%)	17 (24.6%)
Never	19 (61.3%)	20 (52.6%)	39 (56.5%)
Unknown	3 (9.7%)	10 (26.3%)	13 (18.8%)
*Age (years)*			
Median [Min, Max]	54.0 [37.0, 82.0]	52.5 [26.0, 90.0]	53.0 [26.0, 90.0]
Stage			
IIIB	1 (3.2%)	1 (2.6%)	2 (2.9%)
IIIC	1 (3.2%)	0 (0%)	1 (1.4%)
IV	29 (93.5%)	37 (97.4%)	66 (95.7%)
*Tumor histology*			
Adenocarcinoma	30 (96.8%)	36 (94.7%)	66 (95.7%)
Adenosquamous	1 (3.2%)	1 (2.6%)	2 (2.9%)
Squamous	0 (0%)	1 (2.6%)	1 (1.4%)
*RET* *fusion partner*			
CCDC6	6 (19.4%)	4 (10.5%)	10 (14.5%)
KIF5B	23 (74.2%)	25 (65.8%)	48 (69.6%)
Other	2 (6.4%)	6 (15.8%)	8 (11.6%)
Unknown	0 (0%)	3 (7.9%)	3 (4.3%)
*Selective RET inhibitor*			
none	31 (100%)	0 (0%)	31 (44.9%)
Pralsetinib	0 (0%)	33 (86.8%)	33 (47.8%)
Selpercatinib	0 (0%)	5 (13.2%)	5 (7.2%)
*RET inhibitor line*			
1st	0 (0%)	15 (39.5%)	15 (21.7%)
2nd	0 (0%)	12 (31.6%)	12 (17.4%)
3rd or more	0 (0%)	11 (28.9%)	11 (15.9%)
*RET inhibitor_prior MKI*			
No	0 (0%)	32 (84.2%)	32 (46.4%)
Yes	0 (0%)	6 (15.8%)	6 (8.7%)
*RET inhibitor_prior ICI*			
No	0 (0%)	28 (73.7%)	28 (40.6%)
Yes	0 (0%)	10 (26.3%)	10 (14.5%)

TKI, tyrosine kinase inhibitor; MKI, multikinase inhibitor with activity against RET; ICI, immune checkpoint inhibitor

### Outcomes and implications of AEs ≥ grade 3

**Fig. 4:**
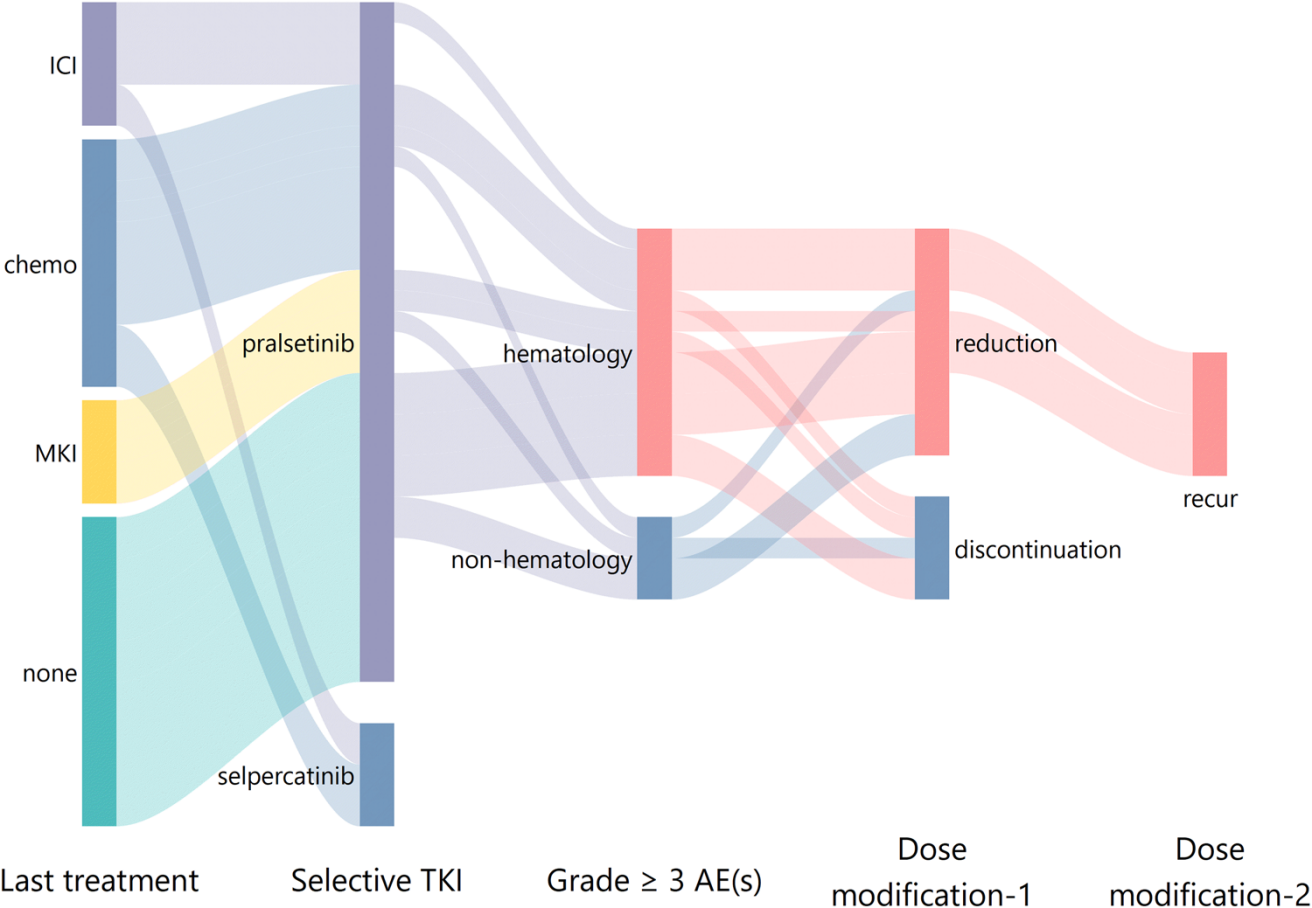
Outcomes of ≥ grade 3 AEs. Plot of treatment information, AEs and outcomes in patients from our study cohort. Abbreviations: TKI, tyrosine kinase inhibitor; AE, adverse event

Of the 38 patients who received selective RET inhibitors from our institute, 16 (42.1%) patients had grade 3 or worse AEs. No correlation was observed between occurrence of AEs ≥ grade 3 occurring and prior immunotherapy/MKI treatment (as the last treatment line received before selective TKI, or once used throughout the course of disease). Median PFS of selective RET inhibition was 11.9 months. There was no significant difference in survival between patients with grade 3 or worse AEs and those without. As shown in Fig. 3C, there was no difference in PFS between patients with or without AEs ≥ grade 3 throughout the course of RET inhibition (median 11.9 versus 12.4 months; HR: 0.92, 95% CI 0.38–2.23, *p* = 0.63). All patients suspended their drug and received timely supportive care, followed by dosage reduction (*n* = 11) or drug discontinuation (*n* = 5). Given that a fraction of our TKI-treated cohort underwent drug discontinuation, we analysed TTF, which takes into account drug discontinuation due to treatment toxicity. TTF did not significantly differ between patients with or without AEs ≥ grade 3 (median 9.3 versus 13.2 months; HR: 1.24, 95% CI 0.53–2.93, *p* = 0.62). Most grade 3 or worse non-haematological AEs like hepatotoxicity resolved after guidance and supportive care, and a large majority of patients were able to resume treatment. However, haematological toxicity (including anaemia, lymphopenia, neutropenia, thrombocytopenia), which was reported in twelve of our patients (31.6%), was the leading cause of drug discontinuation. It was recurrently observed in six patients underwent dosage reduction (Fig. 4).

### Impact of clinical indicators on survival outcomes of selective RET inhibitors

**Fig. 5:**
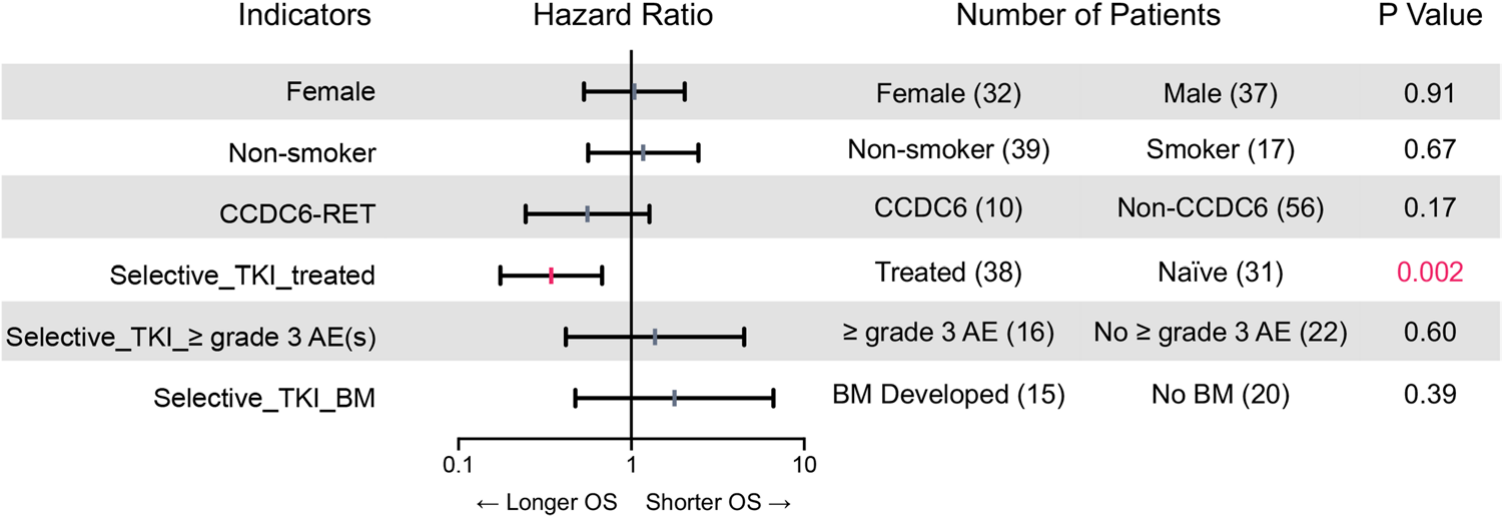
Impact of different clinical factors on survival outcomes of *RET* fusion-positive NSCLC patients. Forest plots of the impact of clinicopathological features, treatment regimens and brain metastasis throughout the course of disease on overall survival. Abbreviations: TKI, tyrosine kinase inhibitor; AE, adverse event; BM, brain metastases

Having established that selective RET inhibitors were being increasingly adopted into clinical practice and were associated with improved overall survival, we further looked into the impact of clinical indicators on survival outcomes of selective RET inhibitors. The impact of clinicopathological features and treatment regimens throughout the course of disease on overall survival are illustrated in Fig. 5. Smoking history and fusion partner had no impact on overall survival of patients treated with selective RET TKIs. There was no difference in overall survival between patients with and without brain metastases (pre-existing or developed during treatment). Notably, of patients with disease progression and known progression sites, the frequency of intracranial disease progression was 23.5% (4/17), with most lesions already existing at baseline. Patients who were treated with RET TKIs had a shorter, albeit not significant PFS in case of intracranial progression (median 4.8 months versus 9.0 months for extracranial progression; HR: 1.97, 95% CI 0.50–7.77, *p* = 0.334) and a shorter TTF (median 5.7 months versus 7.0 months for extracranial progression; HR: 2.14, 95% CI 0.52–8.71, *p* = 0.289).

## Discussion

This study reveals the increasing application of selective RET inhibitors in clinical practice, with patients treated with RET inhibitors having prolonged overall survival compared with selective TKI-naive patients. During the study period, rates of chemotherapy and MKIs decreased whereas rates of selective RET inhibitors in the first-/second line treatment increased. Besides, we observed grade 3 or worse haematologic AEs occurred frequently in real-world setting. It was the leading cause of drug discontinuation and was often recurrently observed in patients underwent dosage reduction.

Our findings are meaningful and have important clinical implications for the selection of systemic treatment strategies in *RET*-driven patients. Phase I/II trials of pralsetinib and selpercatinib revealed a high ORR in both pretreated (61% and 64%, respectively) and untreated (70% and 85%, respectively) patients. Interestingly, the responses were independent of prior treatment received (chemotherapy, immune checkpoint blockade or MKIs) and fusion partner (Gainor et al. 2021; Drilon et al. 2020). Currently, there are several clinical trials comparing the efficacy of selective inhibitor and immunotherapy—both standard of care in this subset of patients (NCT04222972, NCT04194944, and NCT03906331). However, recognizing that follow up was yet to mature, the overall survival of selective RET TKI in trial-setting has not been reported. Our findings indicated that exposure to selective drugs throughout the course of disease was associated with superior overall survival after a median follow up time of 28.7 months. But there was no difference in exposure to ICIs or not. These findings were consistent with results from a study by Tan et al. (2020), with larger sample size and longer follow-up time. Furthermore, results from our previous study (Lu et al. 2020) and a retrospective analysis from Offin et al. (2019) have consistently indicated that *RET*-rearranged NSCLC may respond poorly to immunotherapy, largely secondary to low PD-L1 expression and low TMB. Our results suggested that the presence of *RET* fusion should be determined, and that selective inhibitor should be given priority before administering ICIs.

In addition to the relatively large sample size and long follow-up time, our study has several other key strengths. The study of ordinary patients in the real-world setting guarantees the generalizability of our results. Frequency of grade 3 or worse treatment-related AEs (42%) in our study cohort was comparable to that of the NSCLC population of the LIBRETTO-1 (28%; Drilon et al. 2020) and ARROW (48%; Gainor et al. 2021) study. Findings from the ARROW trial demonstrated that the most common grade 3 or worse treatment-related AEs in NSCLC patients were neutropenia (18%), hypertension (11%), and anaemia (10%) (Gainor et al. 2021; Ali et al. 2022); while in LIBRETTO-001 trial, the most common treatment-related AEs of grade 3 or 4 were hypertension (9%), increased ALT (9%), and increased AST level (6%)(Drilon et al. 2020). In preclinical models, both selpercatinib and pralsetinib showed potent selective activity against RET with significantly diminished affinity for VEGFR2 (> 100-fold and 87-fold selectivity against VEGFR2, respectively), highlighting that off-target inhibition is minimized (Brandhuber et al. 2016; Ali et al. 2022). Although VEGFR2/KDR-related AEs ≥ grade 3 (skin toxicity and proteinuria) were not observed in treatment course of selective TKI due to their lower activity against VEGFR compared with MKIs (Subbiah et al. 2020), ≥ grade 3 haematologic toxicity and hepatotoxicity were common. Moreover, a substantial portion of our patients underwent dosage reduction, drug interruption or discontinuation, but no correlation was observed between the occurrence of AEs and prior immunotherapy/MKI treatment. Grade 3 or worse AEs were mainly manageable with timely supportive care, without having an impact on survival. Given the increasing utilization of selective RET inhibitors in lung cancer patients, these findings call for awareness and guidance of toxicity before initiating treatment with these drugs, as well as monitoring and timely management of AEs during long-term use.

It is worth noting that haematological AEs occurred in 31.6% of our patients. Haematological toxicity was also the leading cause of drug discontinuation since it recurred even after dosage reduction, mostly in patients treated with pralsetinib. This was probably a consequence of RET inhibition, since RET are reported to play a role on haematopoietic stem cell survival and function (Fonseca-Pereira et al. 2014). To date, there is limited data on frequency of treatment-related haematological toxicity of selpercatinib and pralsetinib, since previous reports focused on specific AEs. Our analysis generated from our less-selected real-world study cohort when compared with the clinical study population. In clinical practice, criteria for initiating treatment with selective RET TKI were less strict in baseline laboratory test results and did not follow the window of 14 days required in a clinical trial. Overall, real-world data analyses are generating meaningful data about the safety information on treatment in routine clinical practice.

In addition to toxicity, emergence of resistance to TKIs, which can be anticipated in all treated patients, also prevent a durable response. Brain metastases represent another challenging scenario for personalized treatment. Nearly a quarter of the patients (23.5%) on selpercatinib and pralsetinib in our study cohort had intracranial disease progression. It is worth noting that after the date of data cut-off, two patients in our study cohort also had intracranial disease progression. This relatively high frequency is consistent with a retrospective analysis by Lin et al. (2020), in which 28% patients had intracranial disease progression. Although the two agents have impressive central nervous system activity (Gainor et al. 2021; Subbiah et al. 2021), we observed a numerically shorter PFS of RET inhibitor in patients with intracranial disease progression. However, most of the mechanisms of resistance to pralsetinb and selpercatinib are reported to be off-target (Fancelli et al. 2021), limited the strategy of subsequent treatments. Those results about real-world toxicities and intracranial treatment failure reflect an urgent need for the development of next-generation TKI with less toxicity to ensure administration of adequate drug doses, and more robust brain penetration to prevent or delay intracranial treatment failure, and ultimately improve survival outcomes.

This study has several limitations. First, the impact of each clinical indicator on survival outcomes was determined using univariate analysis performed on a limited sample size and need to be validated in future trials. Limitations also include the variable clinical routine practices performed in real-world settings, in terms of intervals of tumour assessments and safety follow-up visits, and the lack of a formal case report form with all safety variables tabulated for each patient. Moreover, reporting bias and information bias of AEs, especially AEs in non-routinely performed examination (e.g. urinalysis, electrocardiogram), could possibly lead to under-reporting of toxicities. Nevertheless, our findings are based on real-world evidence from patients treated at the same centre using the same workup. Thus, our findings give important insights into personalized treatment.

## Conclusions

This study provides evidence on the increasing application and superior survival outcomes of selective RET inhibitors in *RET*-driven NSCLC patients. Grade 3 or worse AEs, especially haematologic AEs, occurred frequently in real-world setting. Haematologic AEs was the leading cause of drug discontinuation and was often recurrently observed in patients underwent dosage reduction. These findings provide important reference to both clinicians and patients in treatment selection and call for awareness of toxicity during long-term use.
